# Supplementary figures and images for: Cytoplasmic RRM1 activation as an acute response to gemcitabine treatment is involved in drug resistance of pancreatic cancer cells
Source: PLoS One. 2021 Jun 10;16(6):e0252917. doi: 10.1371/journal.pone.0252917 (PMC8191885; doi:10.1371/journal.pone.0252917)

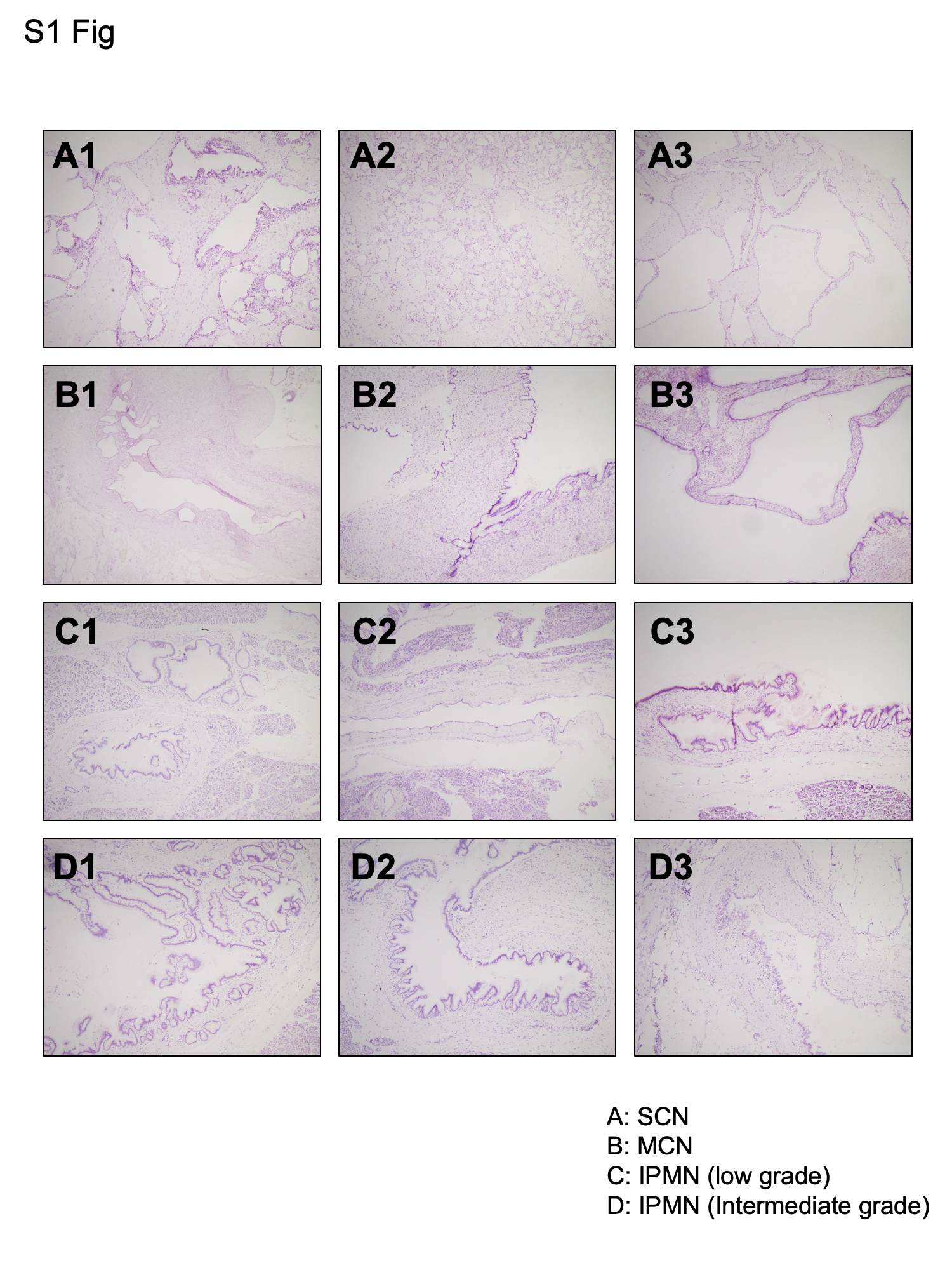

Supplement: S1 Fig — (A) Serous cyst neoplasia (SCN), (B) mucinous cyst neoplasia (MCN), (C) intraductal papillary mucinous neoplasia (IPMN) with low-grade dysplasia, and (D) IPMN with intermediate-grade dysplasia. No RRM1 expression was detected in all benign cases without malignant potential. (TIF) [file pone.0252917.s001.tif]

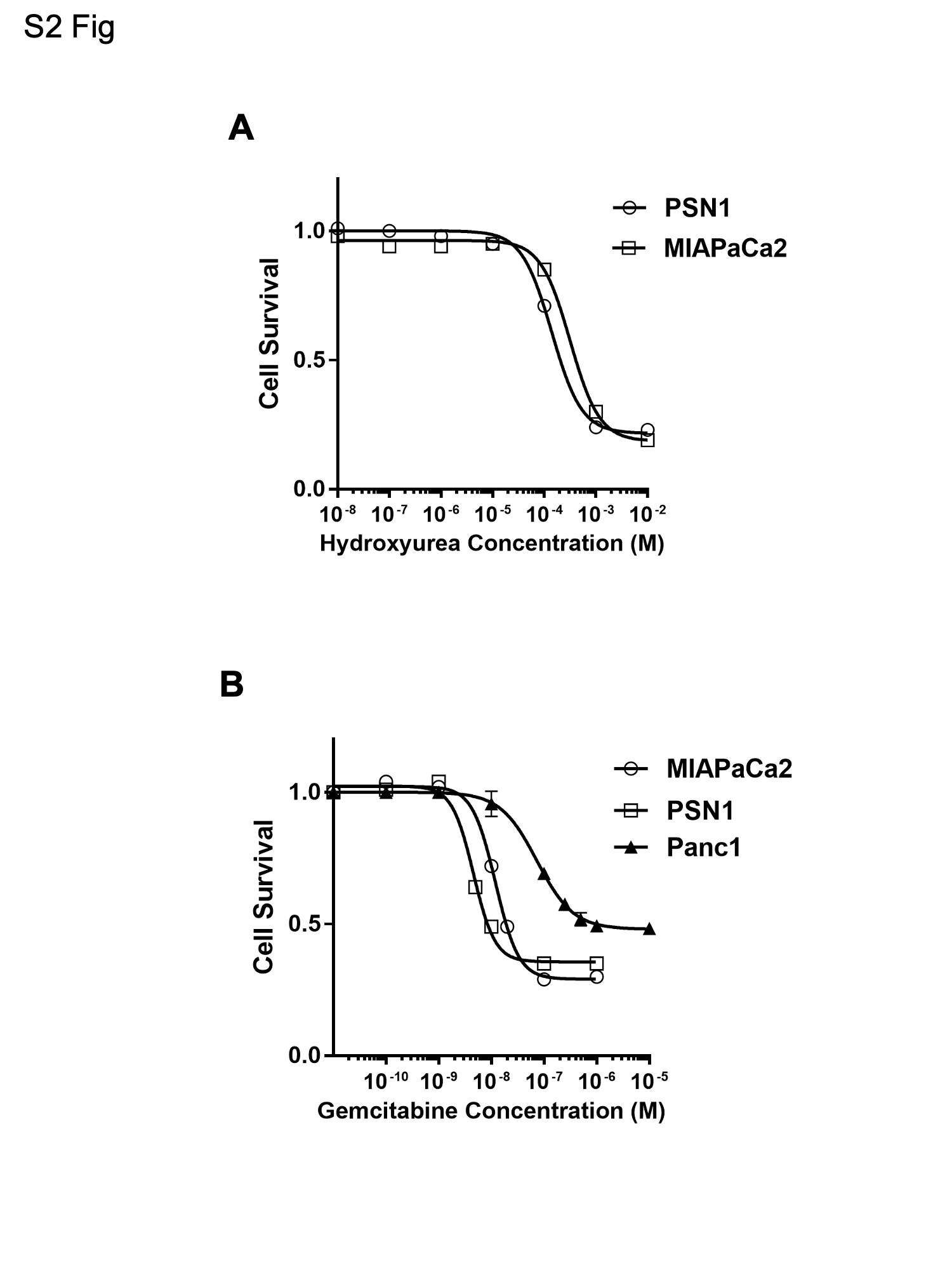

Supplement: S2 Fig — (A), (B) Cells (5–7.5×10^3^ per well) were seeded and incubated overnight. Then each concentration of hydroxyurea or gemcitabine was administrated for 72 hours. (A) Cell viability analysis following hydroxyurea treatment in PSN1 and MIAPaCa2 cells (10 nM-10 mM). IC50 concentration for hydroxyurea was calculated at 140 μM for PSN1 and 328 μM for MIAPaCa2 cells. Cell viability was performed by WST-8 assay. (B) Cell viability analysis following gemcitabine treatment in PSN1, MIAPaCa2, and Panc1 cells (PSN1 and MIAPaCa2; 0.1 nM-1 μM, Panc1; 0.1 nM-10 μM). (TIF) [file pone.0252917.s002.tif]

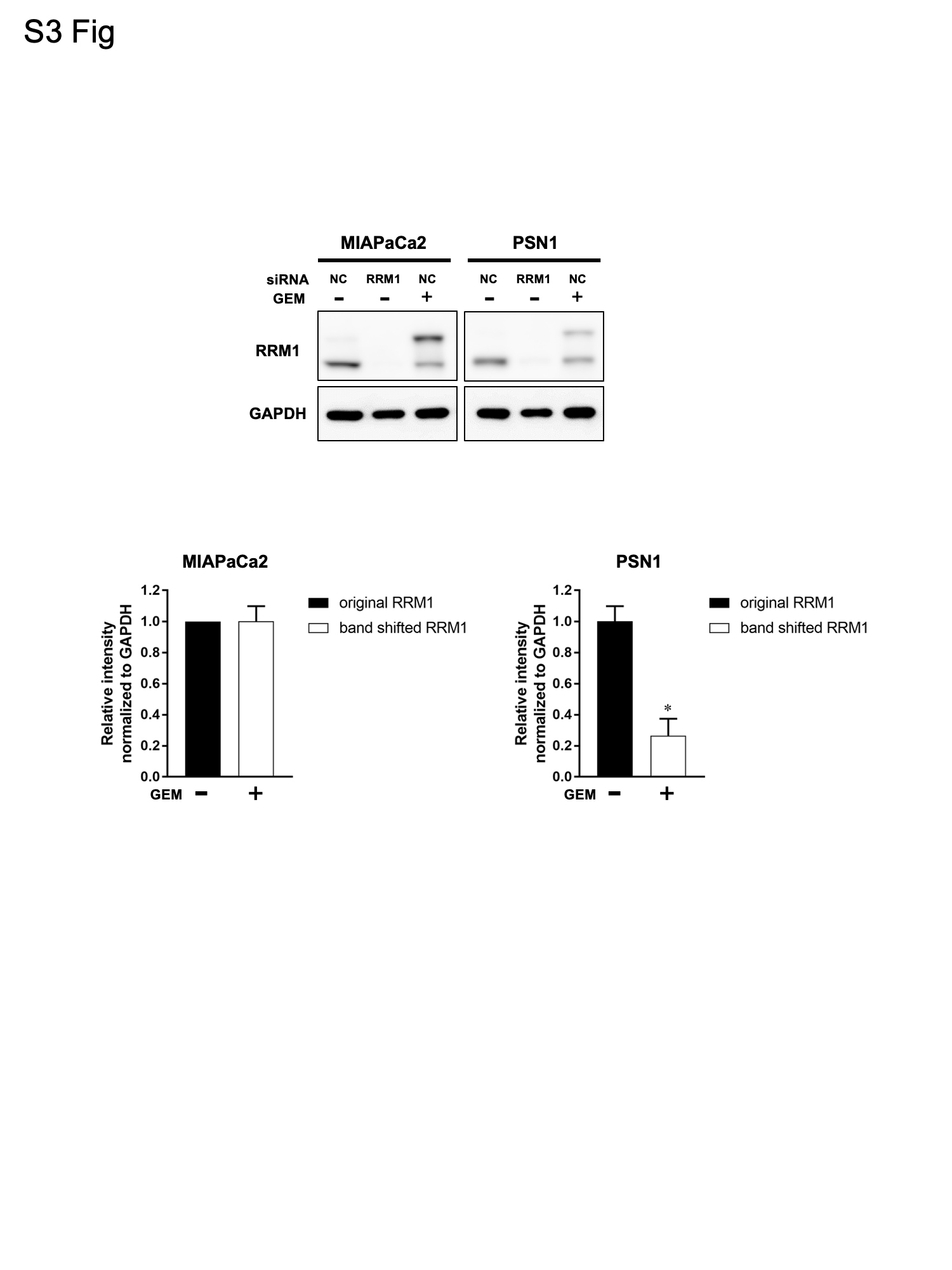

Supplement: S3 Fig — Cancer cells were treated with siRRM1 or siNC for 12 hours. Subsequently gemcitabine was treated for 48 hours (MIAPaCa2; 20 nM and PSN1; 7.5nM). Top, representative Western blotting; bottom, the graph depicts averaged intensity of bands representing band shifted RRM1 expression comparing to original RRM1 intensity without gemcitabine, normalized to the intensity of bands representing GAPDH. Error bars represent mean ± SD. *p<0.05 vs cells with gemcitabine treated MIAPaCa2 cells by unpaired t-test. The Western blotting assay was performed in triplicate. (TIF) [file pone.0252917.s003.tif]

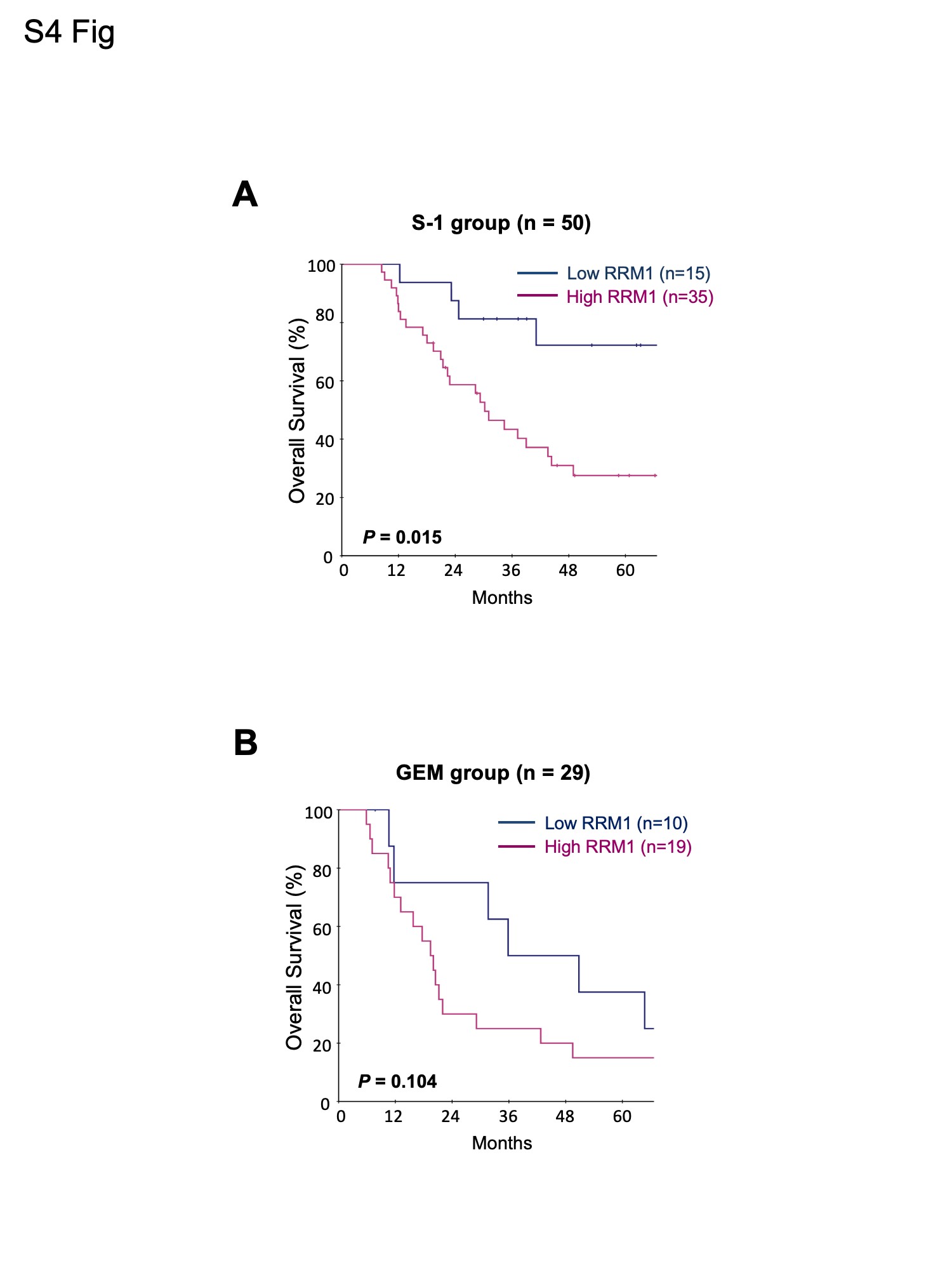

Supplement: S4 Fig — (A) Kaplan-Meier curve for overall survival of patients who received S-1 adjuvant chemotherapy (S-1 group). (B) Kaplan-Meier curve for overall survival of patients who received gemcitabine-based adjuvant chemotherapy (GEM group). (TIF) [file pone.0252917.s004.tif]
