# Supplementary material for: Cytoplasmic RRM1 activation as an acute response to gemcitabine treatment is involved in drug resistance of pancreatic cancer cells
Source: PLoS One. 2021 Jun 10;16(6):e0252917. doi: 10.1371/journal.pone.0252917 (PMC8191885; doi:10.1371/journal.pone.0252917)
Supplement: S1 Raw images — (PDF) [file pone.0252917.s005.pdf]

Fig 3

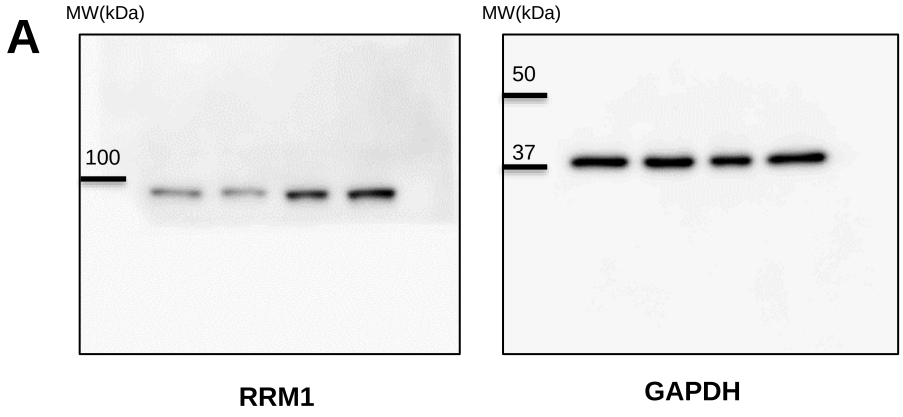

Fig 3A Endogenous RRM1 expression in Hs766T, MIAPaCa2, PSN1, and Panc1 cells. GAPDH was used as internal control.

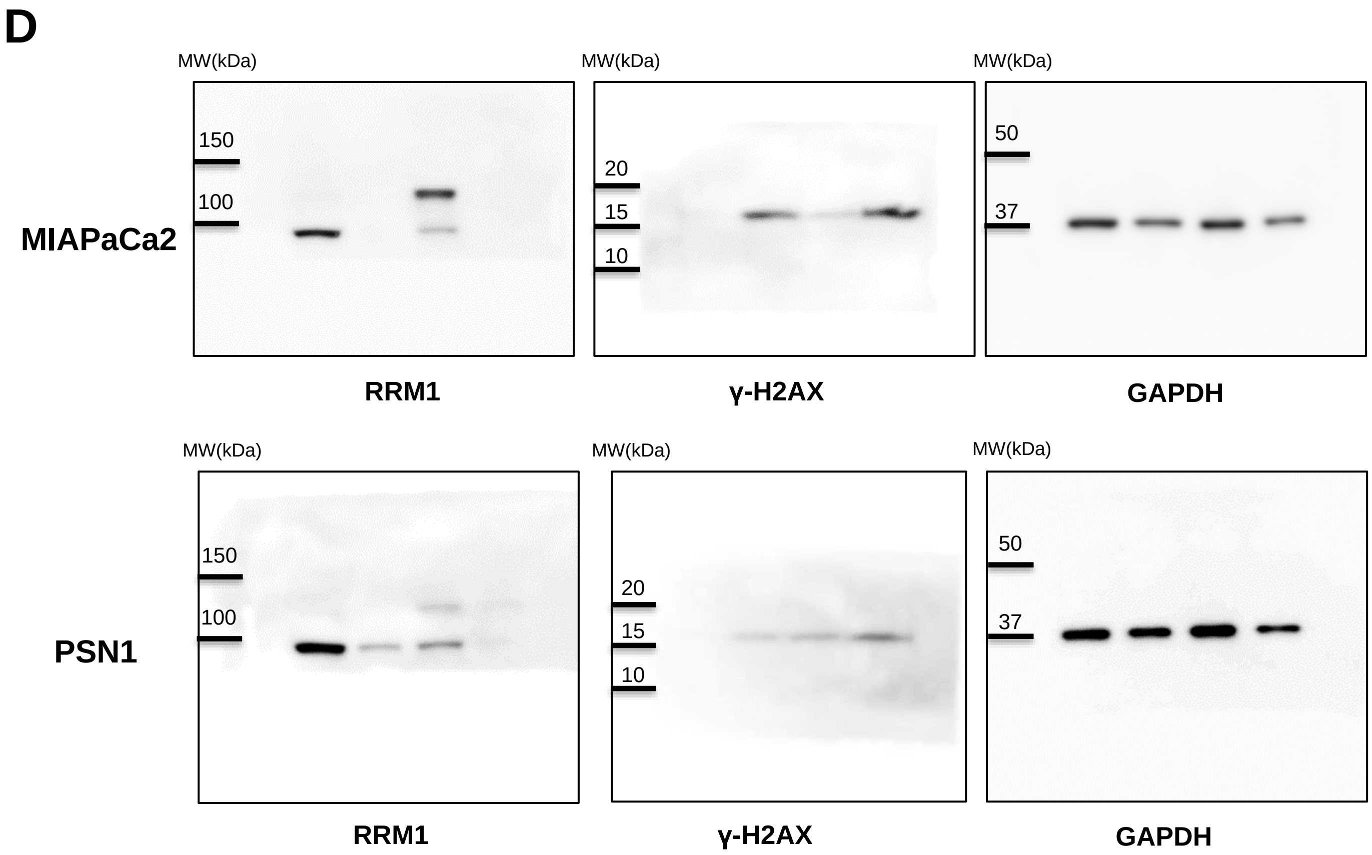

Fig 3D Effects of RRM1 gene-silencing and gemcitabine on RRM1 expression and γ-H2AX expression. 4 lanes: siNC without gemcitabine, siRRM1 without gemcitabine, siNC with gemcitabine, siRRM1 with gemcitabine, Top, MIAPaCa2, bottom PSN1 cells. GAPDH was used as internal control.

Fig 3

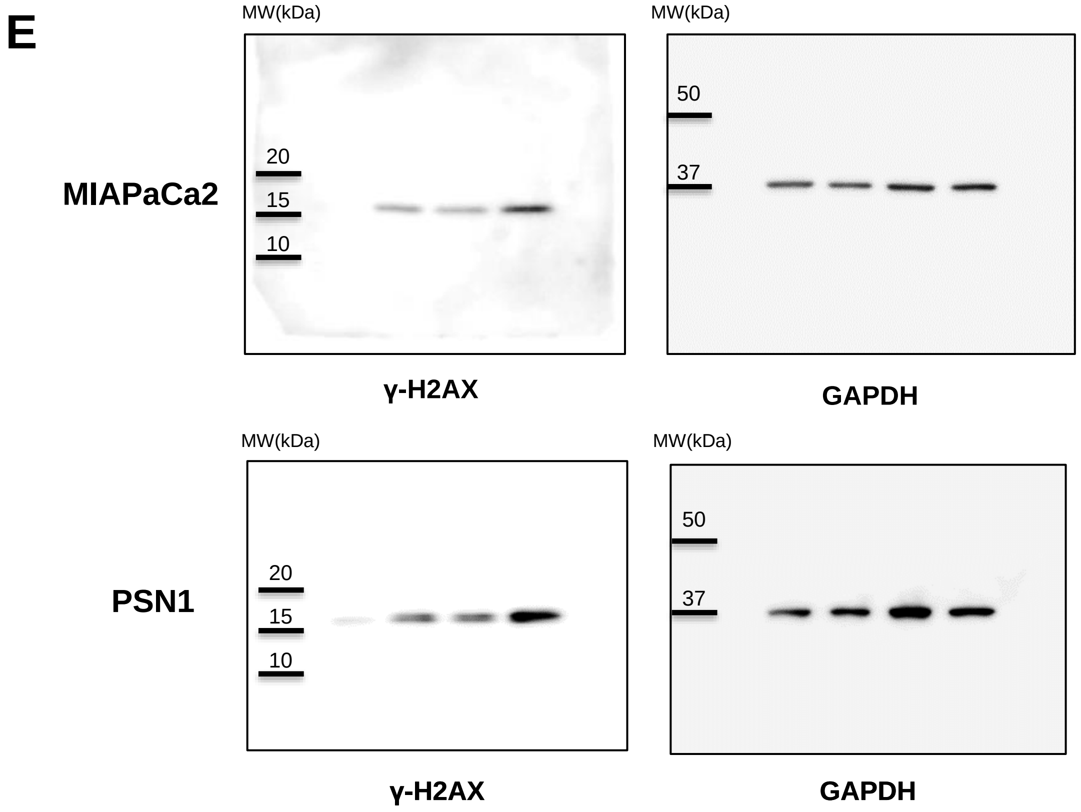

Fig 3E Effects of hydroxyurea(HU), gemcitabine(GEM), and combination with HU and GEM on γ-H2AX expression. 4 lanes: non-treatment, HU, GEM, HU and GEM
Top, MIAPaCa2, bottom PSN1 cells. GAPDH was used as internal control.

Fig 4

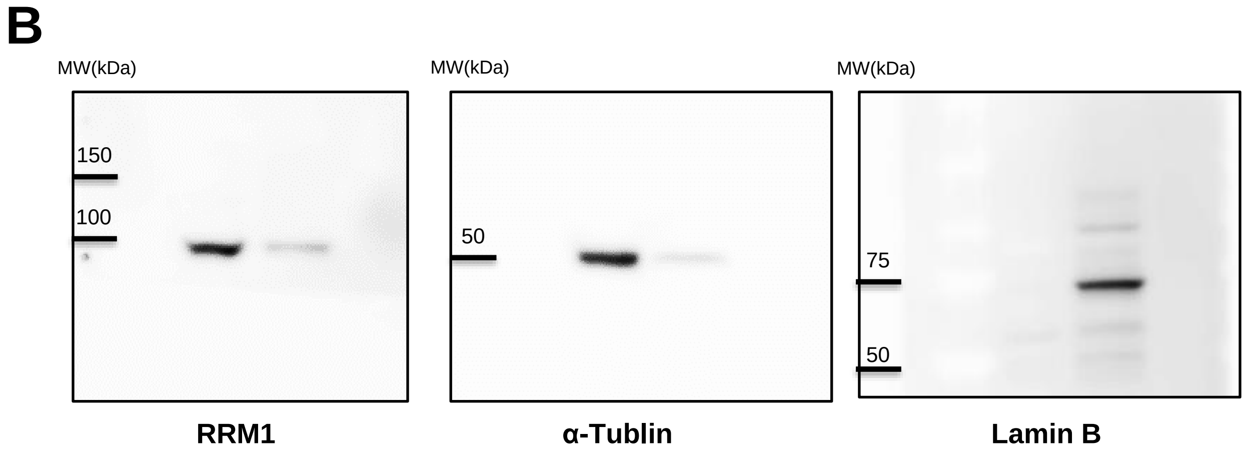

Fig 4B RRM1 expression in nuclear and cytoplasm fraction in Panc1 cells.
2 lanes; left/cytoplasm, right/nucleus. α-Tublin and Lamin B were used as internal control of cytoplasm and nucleus.

Fig 4

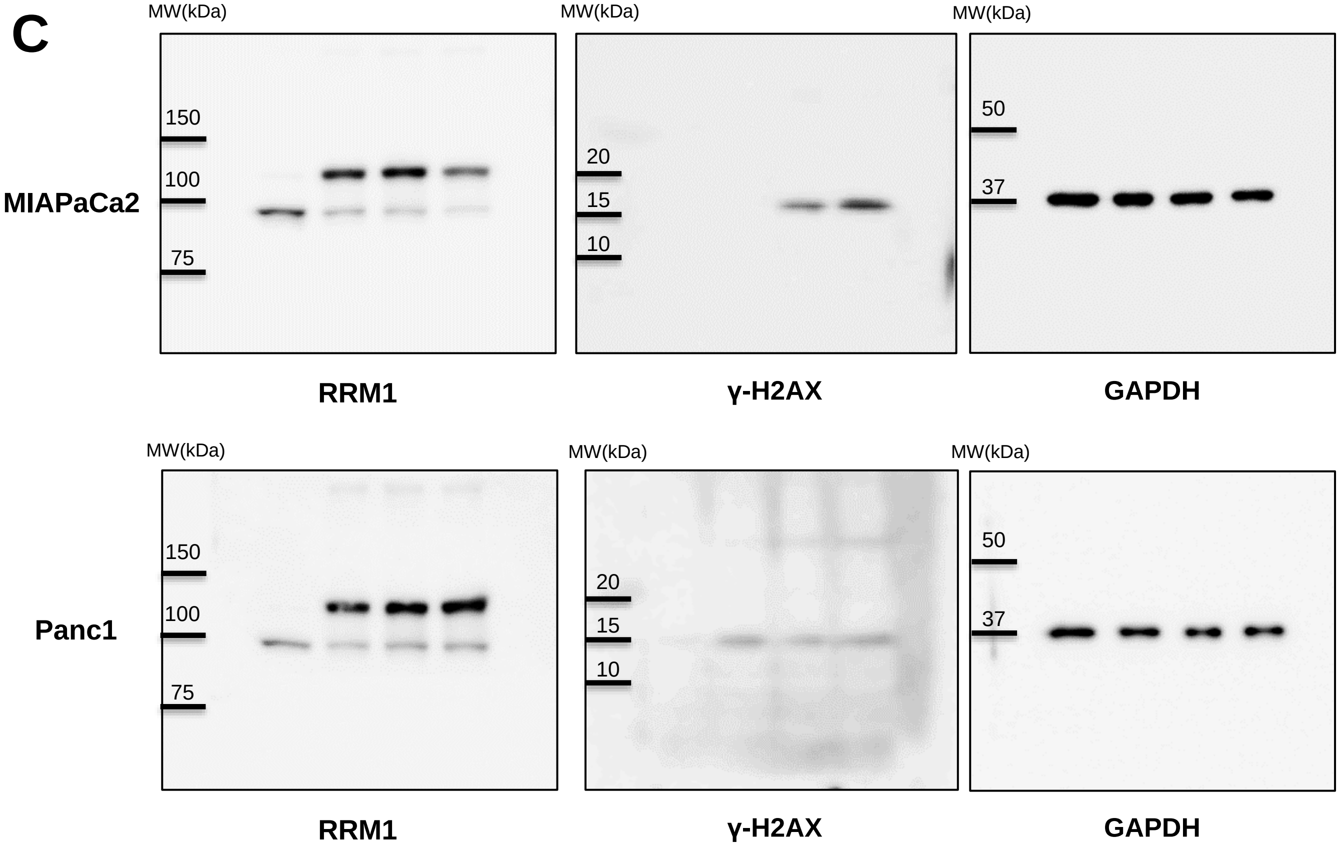

Fig 4C RRM1 and γ-H2AX expression after gemcitabine exposure from 24 hours to 72 hours. 4 lanes: 0, 24, 48, and 72 hours after GEM treatment.
Top, MIAPaCa2, bottom Panc1 cells. GAPDH was used as internal control.

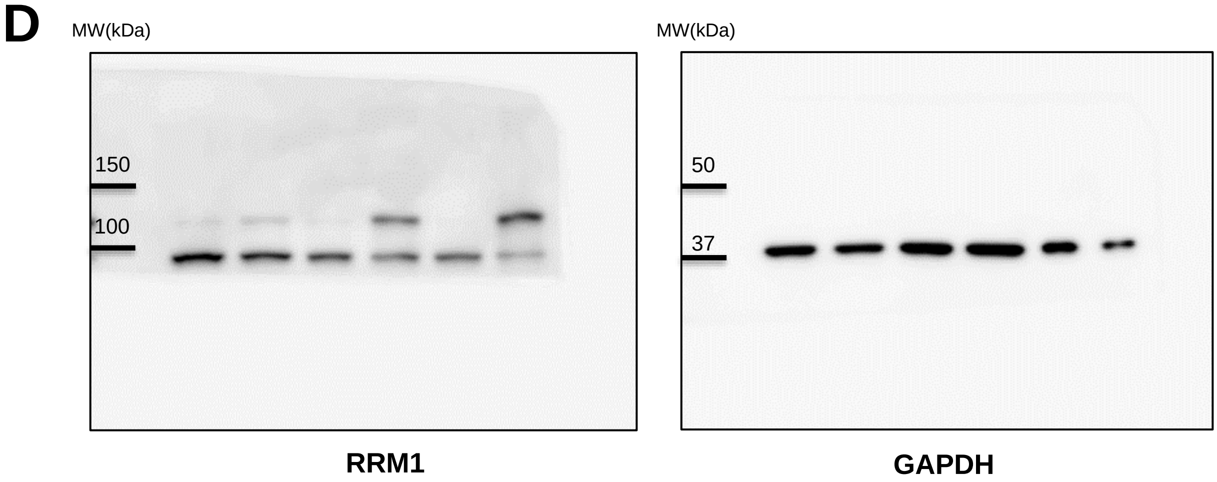

Fig 4D Effects of gemcitabine treatment on RRM1 expression in PSN1, MIAPaCa2, and Panc1 cells. 6 lanes: non-treated PSN1, GEM PSN1, non-treated MIAPaCa2, GEM MIAPaCa2, non-treated Panc1, GEM Panc1. GAPDH was used as internal control.

Fig 4

E

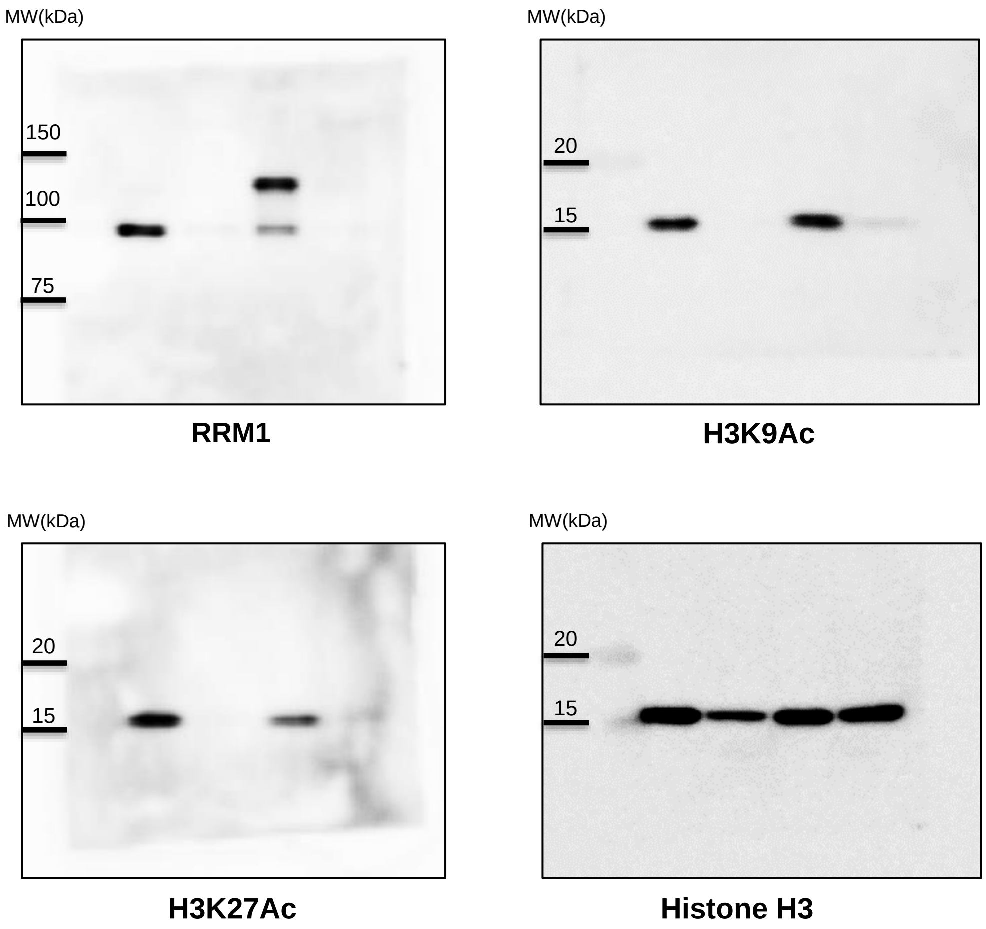

Fig 4E Effects of C646 and gemcitabine treatment on expression levels of RRM1, H3K9Ac, and H3K27Ac in MIAPaCa2 cells. Histone H3 was used as internal control.

Fig 5

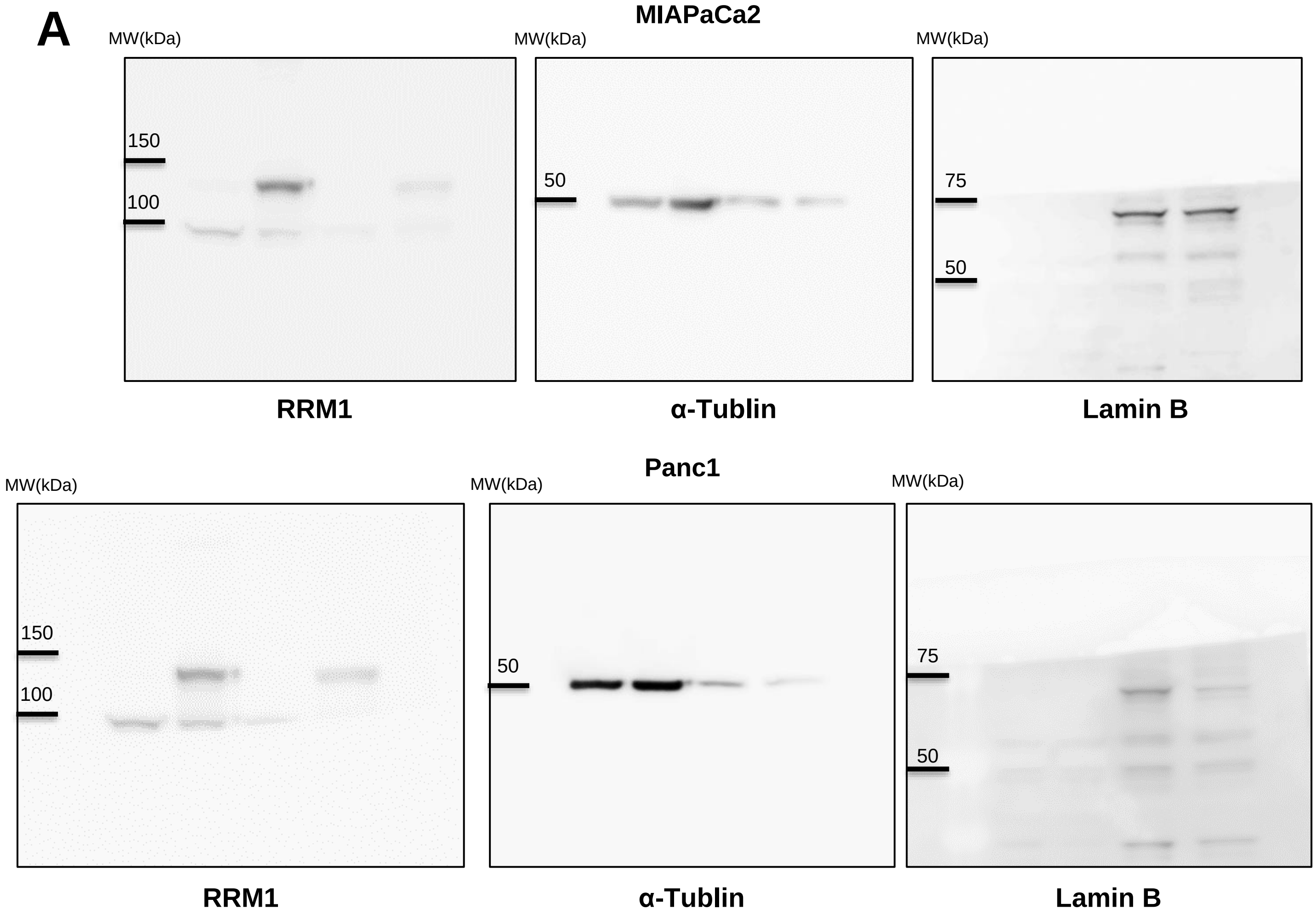

Fig 5A Effects of gemcitabine(GEM) on RRM1 expression levels in cytoplasmic and nuclear fraction of MIAPaCa2 and Panc1 cells. 4 lanes: non-treated cytoplasm, GEM cytoplasm, non-treated nucleus, GEM nucleus. α-Tublin and Lamin B were used as internal control of cytoplasm and nucleus. Top, MIAPaCa2, bottom Panc1 cells.

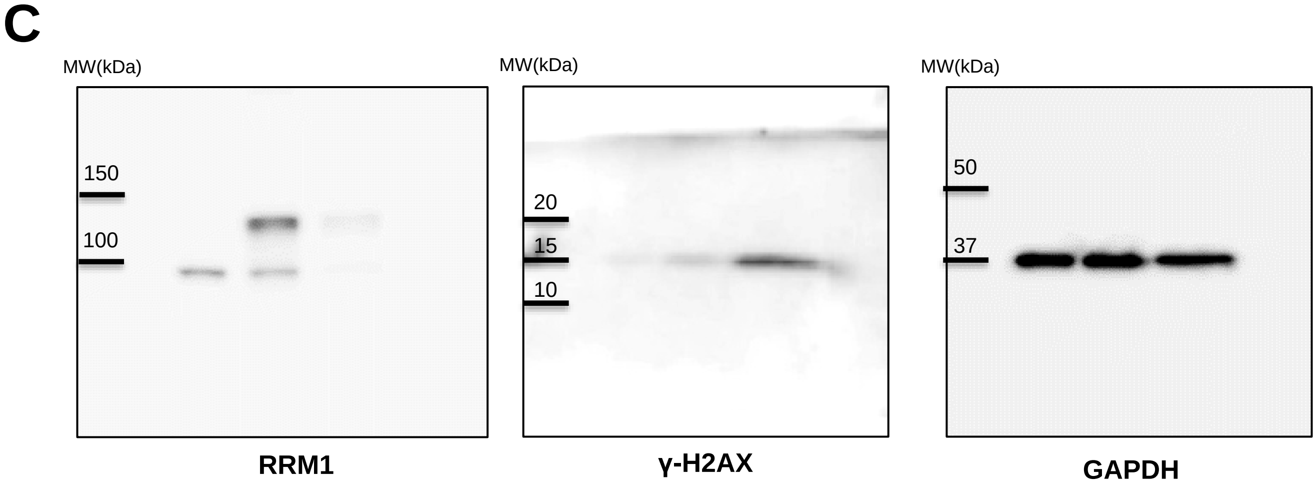

Fig 5C Panc1 cells were fractionated into attached and free-floating cells after gemcitabine treatment. 3 lanes: non-treatment, gemcitabine-attached fraction, gemcitabine-free floating fraction. GAPDH was used as internal control.

S3 Fig

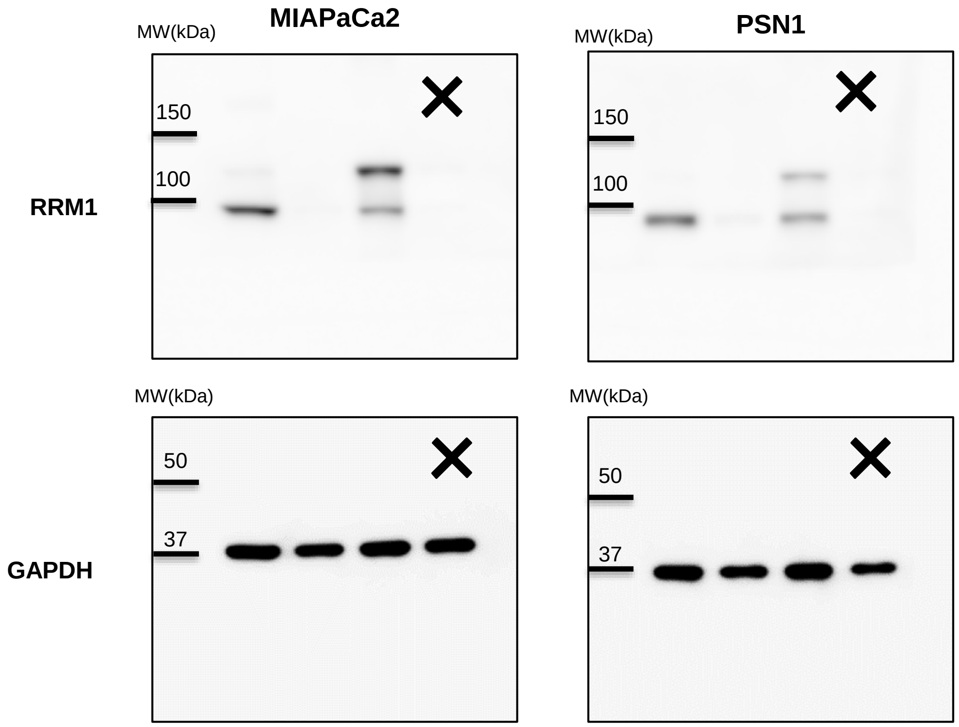

S3 Fig siRRM1 or siNC treated MIAPaCa2 and PSN1 cells were treated with or without gemcitabine (GEM). 4 lanes: non-treated siNC, non-treated siRRM1, GEM siNC, GEM siRRM1. Left, MIAPaCa2, right, PSN1 cells. Last lane (GEM-siRRM1) was not presented in this S3 Fig.
