# Supplementary material for: Cytoplasmic RRM1 activation as an acute response to gemcitabine treatment is involved in drug resistance of pancreatic cancer cells
Source: PLoS One. 2021 Jun 10;16(6):e0252917. doi: 10.1371/journal.pone.0252917 (PMC8191885; doi:10.1371/journal.pone.0252917)
Supplement: Attachment — Submitted filename: Response to Reviewers.docx [file pone.0252917.s006.docx]

Irina V. Lebedeva, Ph.D.

Academic Editor

PLOS ONE

Dear Dr. Lebedeva,

We would like to thank all the reviewers of our manuscript for their insightful comments and helpful suggestions. We have added new data and revised the manuscript accordingly. Each of the issues raised by the reviewers is addressed specifically below.

**Reviewer #1:**

*1. The role of the expression in RRM1 in survival of pancreatic cancer is less controversial than they present. Please see publications by Sierzega et al, Pancreas 2017,* *and the meta-analysis of Wei et al, Pancreas 2013.*

**Comments:** Thank you for your valuable opinion. We agree with reviewer’s comments about the description for the clinical significance of RRM1 expression. The reports by Wei et al (Pancreas 2013) and Sierzega et al (Pancreas 2017) were referred to in our manuscript and we revised our statement in the introduction section described as below．

“RRM1 has also been reported to affect disease prognosis of pancreatic cancer. In fact, the meta-analysis for RRM1 demonstrated that high expression of RRM1 was significantly associated with worse overall survival. The clinical impact of RRM1 has been elucidated. However, the functions of RRM1 in pancreatic cancer biology remain unclear.”

*2. The authors refer to RRM1 activation as part of the acquired resistance to gemcitabine. This is not the proper use of the concept. Acquired resistance would be evolution or selection of a subclone that is resistant to gemcitabine after exposure. Their investigation reports inherent or de novo resistance.*

**Comments:** Thank you for your important advice. As the reviewer pointed out, we have modified acquired resistance to drug resistance in our title and manuscript.

*3. The authors need to provide information on the quantification of the IHC and how they set cutoff points for "high" and "low" expression.*

**Comments:** According to reviewer’s suggestion, we revised our statement in the Figure Legends section to clarify the definitions for staining of RRM1 expression. We added the following sentence in the Figure Legends section.

“For the immunohistochemical staining of RRM1 in pancreatic cancer, the islets of Langerhans served as a positive internal control to evaluate the expression. RRM1 expression was graded into RRM1 expression levels as low (no staining or weak intensity staining comparing to internal control in less than 30% of cells) or high (strong intensity staining in more than 30% of cells based on cytoplasmic staining intensity). The staining grade of RRM1 expression was assessed by two investigators and reviewed by one pathologist.”

*4. The data related to RRM1 expression and resistance to chemotherapy (Figure 2C, C) would be better presented if they compared the RRM1 high group treated with and without gemcitabine, anticipating no benefit of gemcitabine, and comparing the RRM1 low group treated with and without gemcitabine in which a benefit was observed.*

**Comments:** We agree with the reviewer’s suggestion. As reviewer pointed out, we revised the data related to RRM1 expression and resistance to chemotherapy in Figure 2C and 2D. Figure 2C and 2D represent the clinical efficacy of RRM1 expression on a benefit of adjuvant chemotherapy comparing low and high RRM1 expression in the revised manuscript. Thanks to the reviewer’s valuable advice, our assertion concerning the biological role of RRM1 on clinical benefit of adjuvant therapy has been clarified.

*5. The Results section of the manuscript around the data of Figure 3 is very confusing. They refer to Figure 3B in the text, but it seems like they are referring to Figure 3C (Figure 3B is the reduced expression of RRM1 after siRNA treatment). In addition, there are no Figure legends for Figures 3D and 3E.*

**Comments:** We appreciate the reviewer’s meticulous review in the very detail point. The Result section of Figure 3B and 3C and corresponding Figure legends have been changed appropriately. And we have added Figure legends for Figure 3D and 3E in our revised manuscript. We apologize for this careless mistake in our figures and figure legends and have corrected this in the revision.

*6. The data presented in Figure 3E is difficult to interpret. How does the data show increased DNA damage accumulation.*

**Comments:** According to reviewer’s suggestion, the experiment was re-performed for western blotting to clarify increased DNA damage accumulation in MIAPaCa2 and PSN1 cells after hydroxyurea and gemcitabine treatment.

*7. The investigation that cytoplasmic activation of RRM1 is related to the baseline levels of RRM1? Namely for those cells with high baseline, do they demonstrate the cytoplasmic activation to the same extent as those with low RRM1? The data of Figure 3A shows some variation in baseline RRM1 in which HS766T may be worth investigating.*

**Comments:** We really appreciate for your raising an important question and providing these insights. Since it is especially important on this point in the manuscript, we have performed some additional experiments. In our opinion, cytoplasmic activation of RRM1 is not related to the baseline level of RRM1 expression. We rather speculated that this activation is related in response to DNA damage repair in pancreatic cancer cells. In fact, the basal RRM1 expression in PSN1 cells was higher than MIAPaCa2 cells as shown in Figure 3A. Whereas, RRM1 alteration after gemcitabine exposure was significantly scant in PSN1 cells, which are gemcitabine-sensitive comparing to MIAPaCa2 cells as shown in the newly added Supplementary Figure S3 and Figure 4. In addition, cross-sectional analysis of pancreatic cancer cell lines has been added to the revised manuscript as shown Figure 4D. Among pancreatic cancer cell lines we tested, Panc1 cells exhibited the most resistant phenotype to gemcitabine (Supplementary Figure S3). The results of these experiments have supported our notion very well indeed. These results are added in Figure 4D and Supplementary Figure S3 and S4. The relevant parts of Results and Figure Legends sections have been revised accordingly.

**Reviewer #2:**

*#1 Protein expression in primary tissue by IHC is subjected to interpretation by pathologists. Please add detail in the method for who did the grading and whether there was more than one pathologist. Are they reviewing the staining together or separately and how they rectified varying expression in the same tissue and if the pathologists do not agree on the staining intensity.*

**Comments:** Thank you for your valuable suggestion. In this study, two investigators assessed the staining grade of RRM1 expression, and one pathologist judged and reviewed the results separately. For the immunohistochemical staining of RRM1 in pancreatic cancer, the islets of Langerhans served as a positive internal control to evaluate the expression. The grading of RRM1 protein expression in primary tissues was determined into two grades. Tumor cells which had stronger intensity staining comparing to positive control in more 30% of cells were defined as high RRM1 expression. Whereas low RRM1 expression was defined as no staining or weak intensity staining in less 30% of cells. The staining grade of RRM1 expression was assessed by two investigators and the pathologist agreed with determination of the grade and the criteria of RRM1 expression. We revised our statement in the Figure Legends section to clarify the definitions for staining of RRM1 expression.

*#2 Pancreas cancer treatment has improved, so a specific period between the first and last cases need to be included.*

**Comments:** We agree with reviewer’s opinion. We revised our statement with a specific period and relevant parts of the Material and Methods were revised accordingly.

*#3 Please clarify the non-GEM group for what adjuvants these patients received. It is important to know whether they received DNA targeting agents since high RRM1 seems to also be associated with poor survival in this group. Even though there is a trend towards worse survival in the GEM group, the data is not statistically significant. Need to discuss this. Could be a limited sample size but the negative data here does not support further studies of RRM1 and GEM resistance.*

**Comments:** In non-GEM group, S-1 was used as a therapeutic agent for the patients in the postoperative adjuvant setting. S-1 is an oral fluoropyrimidine compound comprising tegafur, gimeracil, and oteracil potassium. S-1 functions as a prodrug of 5-FU and, like gemcitabine, is classified as a DNA targeting agent. The therapeutic use of S-1 as postoperative adjuvant chemotherapy has become the standard of care in Japan. One of the reasons why there was no significant difference in the GEM adjuvant group is the issue about the limited sample size. Another reason is that the overall prognosis in the GEM group was worse than that in the S-1 group, which may indicate that GEM was less effective than S-1 as an adjuvant treatment in addition to the different situation background. In this study, we focused on the biological function of RRM1 against gemcitabine, but the synergistic effect of RRM1 can be expected for S-1, which is also characterized as a DNA-targeted therapeutic agent. The phrase “non-GEM group” was misleading and have corrected. The details about the difference of therapeutic efficacy between gemcitabine and S-1 was described in the Discussion section and the figures has been changed to supplemental materials (Supplemental Figure S2A and S2B). We have accordingly revised the relevant parts of the Results, Figure Legends, and Discussion sections.

*#4 Please discuss the negative PFS with adjuvant therapy but the highly significant OS. This data would suggest an adverse impact of RRM1 in the post relapse setting, unrelated to GEM.*

**Comments:** It has been already common knowledge that GnP (gemcitabine and nab-paclitaxel) or FOLFIRINOX (5-FU, oxaliplatin, and irinotecan) were currently performed for the treatment of pancreatic cancer in the recurrent or metastatic settings. DNA targeting anticancer drugs such as gemcitabine and 5-FU play a central role in the treatment even in the post relapse settings. RRM1 may exert its effect on resistance to multidrug therapy, including DNA-targeted anticancer drugs using after relapse relatively for a long time. Therefore, RRM1 expression probably affects OS rather than DFS in pancreatic cancer patients. A description of these contents has been added to the Discussion section.

*#5 Please describe the siRRM1 cells whether they have different growth rates. This will matter more in your figure 1 when you compare the GEM treatment effects.*

**Comments:** We added the experimental data using siRRM1 cells without gemcitabine exposure in Figure 3B. Cell proliferation was significantly inhibited in siRRM1 cells as well, regardless of basal RRM1 expression. We have accordingly revised the relevant parts of the Results and Figure Legends sections.

*#6 In Figure 3A: please do densitometric analysis and describe the ratio between RRM1 and GAPDH. The MIAPaCa cells look to have the lowest amount of RRM1, so the role of RRM1 is better studied in the overexpression model. PSN is a good model here.*

**Comments:** According to reviewer’s suggestion, we added the data of endogenous RRM1 expression in the four pancreatic cancer cell lines using densitometric analysis as the relative ratio comparing to MIAPaCa2 cells, which possessed the lowest RRM1 expression in our panel as shown in Figure 3A. The relevant parts of the Results and Figure Legends sections have been revised accordingly.

*#7 Figure 3B and C, please add data of siRMM cells without GEM. That will show whether there are any baseline cumulative DNA damage and different growth rate*

**Comments:** As reviewer’s indication, we added the data using siRRM1 cells without gemcitabine exposure to Figure 3B and 3D. Gene-silencing of RRM1 induced DNA damage accumulation and inhibited cell proliferation. These effects against DNA damage accumulation and inhibition of cell viability were synergistically enhanced when gemcitabine was used in combination. The relevant parts of the Results and Figure Legends sections have been revised accordingly.

*#8 Figure 3D: Need HU treatment alone as a control too since HU dose chosen was based on IC50, per author’s description (line 233)*

**Comments:** As reviewer’s indication, we added the data treating with HU alone to Figure 3C and 3E. Like RRM1 gene-silencing, HU monotherapy induced DNA damage accumulation and inhibited cell proliferation. These effects against DNA damage accumulation and inhibition of cell viability were also synergistically enhanced when gemcitabine was used in combination. The relevant parts of the Results and Figure Legends sections have been revised accordingly.

*#9 Fig 4C（D?）showing altered MW of RRM1 with GEM treatment is interesting but C646 decreased both primary and higher MW forms, so cannot use this method to conclude that the altered MW is via acetylation and cannot conclude that acetylation of RRM1 has any significant role in DNA damage. To support this, it would need evidence of a different degree of DNA damage and cell death in GEM +/- C646. The posttranslational modification of RRM1 is an interesting one esp. if it affects the localization and function of the protein. This is a novel finding and needs more expansion of the experiment and/or discussion.*

**Comments:** Previously we reported that a histone acetylation inhibitor C646 increased gemcitabine-induced DNA damage and decreased cell viability in pancreatic cancer cells (Ono H et al, Oncotarget, 2016; 7:51301-5131). Indeed, it is difficult to show a direct relevance between RRM1 acetylation and DNA damage using C646. Since C646 globally represses the transcriptional regulation mediated by downregulation of histone acetylation, therefore it is not suitable for evaluation of simply repression of RRM1 acetylation. We demonstrated that using densitometric analysis, altered molecular weight of RRM1 was significantly inhibited by a histone acetylation inhibitor C646 instead. We agree that the posttranslational modification of RRM1 is an interesting point, but we have not been able to demonstrate the direct relevance of RRM1 acetylation on gemcitabine-induced DNA damage in the revised manuscript. We will leave it as a future research topic. The result was added in Figure 4E in the revised manuscript. The relevant parts of the Results and Figure Legends sections have been revised accordingly.

*#10 Please do densitometry for the ration of cytoplasmic and nuclear RRM1 in Fig 4E. looks like there is an increase in a nuclear fraction too*

**Comments:** As the reviewer mentioned, it appeared that there is also an increase in a nuclear fraction in Panc1 cells. We examined RRM1 expression in the nuclear and cytoplasmic fractions with another cancer cell line using MIAPaCa2 cells. As shown in Figure 5A, in MIAPaCa2 cells, there is a very slight increase in RRM1 expression in the nucleus. Most of band shifted RRM1 alteration occurred in the cytoplasm, whereas the amount of increase in RRM1 nuclear fraction is exceedingly small. At the same time, the band of α-Tublin can be recognized even in the nuclear fraction. Considering these results, we supposed that a partial inclusion of cytoplasmic components is observed due to the limitations of experimental precision rather than an increase in nuclear fractions. A simple comparative analysis of nuclear and cytoplasmic proteins is difficult, so we did not perform a densitometric analysis in this case. The results were added in Figure 5A in the revised manuscript. The relevant parts of the Results and Figure Legends sections have been revised accordingly.

*#11 The description of accumulation of cytoplasmic RRM1 only in cells without DNA damage could be interesting but this needs quantification, rather than just one immunofluorescent staining and should be compared between GEM sensitive and resistant cell line. This point is critical as abnormal translocation of RRM1 and its effect on GEM sensitivity is a novel finding while other clinical roles of RRM1 and its function as determined by siRNA in cell lines have already been previously reported.*

**Comments:** Thank you for your valuable opinion, we completely agree with reviewer’s comments about the necessity of quantification to calculate accumulation of cytoplasmic RRM1 in response to DNA damage. We performed additional investigation for western blotting and subsequent densitometric analysis to examine the RRM1 expression level quantitatively in response to DNA damage by comparing attached on the plate and free-floating cancer cells. As shown in Figure 5C, the quantitative analysis demonstrated that apoptosis-inducing cancer cells abolished RRM1 activation and increased DNA damage accumulation. On the other hand, RRM1 activation in the cytoplasm was confirmed only in attached and viable cells to tolerate gemcitabine-induced DNA damage. We convince that this result quantitatively confirmed the finding that cytoplasmic RRM1 accumulated only in cells without DNA damage. The result was added in Figure 5C and the relevant parts of Results and Figure Legends sections have been revised accordingly.

In conclusion, we would like to thank the reviewers for their careful reading of the manuscript and helpful suggestions. We believe the manuscript is much improved.

Sincerely,

Tomotaka Kato, MD

Hiroaki Ono, MD PhD
