# Supplementary material for: Cytoplasmic RRM1 activation as an acute response to gemcitabine treatment is involved in drug resistance of pancreatic cancer cells
Source: PLoS One. 2021 Jun 10;16(6):e0252917. doi: 10.1371/journal.pone.0252917 (PMC8191885; doi:10.1371/journal.pone.0252917)
Supplement: Attachment — Submitted filename: Response to Reviewers for 2nd revise.docx [file pone.0252917.s007.docx]

Irina V. Lebedeva, Ph.D.

Academic Editor

PLOS ONE

Dear Dr. Lebedeva,

We would like to thank all the reviewers of our manuscript for their insightful comments and helpful suggestions. We have added new data and revised the manuscript accordingly. Each of the issues raised by the reviewers is addressed specifically below.

Review Comments to the Author

Reviewer #3:

*1. For the univariate and multivariate analysis, adjuvant therapy should be one of the variables included in the model, especially the remaining study has shown that adjuvant treatment could have significant survival impact on patients with low RRM1 expression. Thus, adjuvant treatment could be a confounding factor. Thus, this must be included in the multivariate regression model regardless of the P value in the univariate analysis.*

**Comments:**

Thank you for your important advice. As the reviewer pointed out, we added the parameter of the adjuvant treatment in the multivariate analysis in Table 2. It revealed non-adjuvant chemotherapy was one of the independent prognostic factors for overall survival. We revised our manuscript in the Results section accordingly.

*2. Regarding the RRM1 band shift, the data did not clearly support the conclusion regarding the role of histone acetylation. If histone acetylation is responsible directly or indirectly for the upward shift of RRM1, one would expect a stronger bottom band and weaker top band with C646 treatment (fig 4E). Instead, it seems that both bands were lighter. So, histone acetylation does not seem to be responsible for this post-translation modification of RRM1, but perhaps on degradation or expression. It's probably some other protein modifications unrelated to the histone acetylation. How about ubiquitination, parylation, or sumolyation, etc.*

**Comments:**

Thank you for your valuable question. We have repeated the experiment in Figure 4E with the additional administration of C646 monotherapy to address the reviewer’s suggestion. Interestingly, C646 decreased the expression level of RRM1. Although gemcitabine increased the band shift of RRM1, both the band shift after gemcitabine administration and the original expression level of RRM1 were decreased by C646 treatment. These experimental results indicated that RRM1 expression levels and changes in band shifted RRM1 expression are regulated by histone acetylation. Therefore, the relationship between histone acetylation and alteration of RRM1 expression after gemcitabine administration has been well established, and in this manuscript, we focused only on histone acetylation. The other translational modifications such as ubiquitination, parylation, and sumolyation, which were pointed out by the reviewer, are of very interest, however, will be the subject of future experiments. And we have clarified the relationship between RRM1 expression and histone acetylation has been clarified in our other papers under submission. The result is added in Figure 4E. The relevant parts of Results and Figure Legends sections have been revised accordingly.

*3. In their conclusion, the authors stated "RRM1 activation". I am not sure what "activation" did the author refer to. All we have seen was a band shift with no clear mechanism. How do we know the higher molecular weight RRM1 is the active form?*

**Comments:**

We appreciate your insightful suggestion. In this study, we confirmed that RRM1 expression in the cytoplasm was indeed increased in cancer cells acquiring gemcitabine resistance. The Western blot in Figure 4C showed that the band shifted RRM1 expression increased in response to gemcitabine treatment compared to the original band. Figure 5C also showed that the band shifted RRM1 expression was associated with the acquisition of gemcitabine resistance. We showed the results of fluorescence immunostaining in Figure 5B, suggesting that gemcitabine-resistant cancer cells were likely to have increased band shifted RRM1 expression mainly in the cytoplasm. Accordingly, total RRM1 expression combined with original band and band shifted form was activated. It is certainly difficult at this point to determine whether the band shifted RRM1 is in an activated form or not. However, from these experimental results representing Figure 4B and other figures, we can finally conclude that increased total RRM1 expression as RRM1 activation is associated in the acute phase of gemcitabine resistance, regardless of whether it is band shifted or original.

In consideration of the above, we have corrected the manuscript in the Abstract section, without including any definitive expressions that band shift RRM1 expression is an active form after gemcitabine exposure.

In conclusion, we would like to thank the reviewers for their careful reading of the manuscript and helpful suggestions. We believe the manuscript is much improved.

Sincerely yours,

Tomotaka Kato, MD

Hiroaki Ono, MD PhD
